# Efficacy and Resistance Management Potential of a Modified Vip3C Protein for Control of *Spodoptera frugiperda* in Maize

**DOI:** 10.1038/s41598-018-34214-z

**Published:** 2018-11-01

**Authors:** Theodore W. Kahn, Maissa Chakroun, Jayme Williams, Tom Walsh, Bill James, Jessica Monserrate, Juan Ferré

**Affiliations:** 1 BASF Agricultural Solutions Seed US LLC, 3500 Paramount Parkway, Morrisville, NC 27560 USA; 2 ERI de Biotecnología y Biomedicina (BIOTECMED), Department of Genetics, Universitat de València, 46100 Burjassot, Spain; 3 CSIRO, Black Mountain, Clunies Ross St., Acton, 2601 ACT Australia; 4 Present Address: Centre de Biotechnologie de Sfax (CBS), Sfax, Tunisia

## Abstract

A modified Vip3C protein has been developed that has a spectrum of activity that has the potential to be commercially useful for pest control, and shows good efficacy against *Spodoptera frugiperda* in insect bioassays and field trials. For the first time Vip3A and Vip3C proteins have been compared to Cry1 and Cry2 proteins in a complete set of experiments from insect bioassays to competition binding assays to field trials, and the results of these complementary experiments are in agreement with each other. Binding assays with radiolabelled toxins and brush border membrane vesicles from *S*. *frugiperda* and *Helicoverpa armigera* show that the modified Vip3C protein shares binding sites with Vip3A, and does not share sites with Cry1F or Cry2A. In agreement with the resulting binding site model, Vip3A-resistant insects were also cross-resistant to the modified Vip3C protein. Furthermore, maize plants expressing the modified Vip3C protein, but not those expressing Cry1F protein, were protected against Cry1F-resistant *S*. *frugiperda* in field trials.

## Introduction

*Bacillus thuringiensis* is a gram-positive bacterium that produces a variety of insecticidal protein toxins during its different growth phases. Two major categories of insecticidal proteins produced by *B*. *thuringiensis* are delta-endotoxins (Cry and Cyt toxins), that may form crystals within the bacteria during sporulation, and vegetative insecticidal proteins (Vip), which are secreted into the growth medium during vegetative growth. Delta-endotoxins fall into dozens of classes, but all share structural and functional similarities, and all form pores in or otherwise disrupt insect gut brush border membranes^1,2^. Vip proteins fall into four unrelated classes, consisting of Vip1 plus Vip2, which function as a binary toxin, Vip3, and Vip4^3^. The Vip3 class of proteins appear to function by forming pores in insect gut brush border membranes^4^. *Bacillus thuringiensis* pore-forming toxins are believed to interact with receptors in the brush border before forming pores, and the specificity of a given toxin for particular insect species depends largely on the identity of its receptor. The binding of toxins to receptors in the midgut brush border membrane has been shown to play a key role in the mode of action of Cry1 and Cry2 proteins, since many resistant insect strains have been found to have a strong reduction of binding ability^5–11^. In addition, binding analyses using brush border membrane vesicles (BBMV) derived from insect midguts have proved to be a sound method of predicting or explaining patterns of cross-resistance among *B*. *thuringiensis* insecticidal toxins^9,12–17^. However, only a limited number of studies on a limited number of insect species have been carried out with labeled Cry1Fa, Cry2Ae, Vip3Aa or Vip3C to estimate the affinity of these proteins for membrane binding sites and the concentration of these sites in the brush border. Similarly, very few studies have tested the ability of these particular proteins to compete for each other’s binding sites^18–20^. The current study adds to the knowledge in this area, helping to establish more firmly the nature of the interactions of these economically important proteins with their target insects.

In the present study we tested and compared the four proteins listed above in a variety of experiments. We evaluated a modified version of Vip3Ca, called ARP150v02, with improved activity against *Spodoptera frugiperda*, in field efficacy trials, insect bioassays, and brush border membrane binding studies, to explore the extent of cross resistance with commercial Bt proteins. We performed competition binding assays with *S*. *frugiperda* and *Helicoverpa armigera* BBMV, and use these data to propose a binding site model for these insecticidal toxins. Laboratory insect bioassay results and field trial results were consistent with the *in vitro* binding data, suggesting that binding experiments can be used to help predict the resistance management usefulness of an insecticidal protein used as a trait in crops. The fact that the different types of experiments were in agreement with each other will allow for greater confidence in the use of laboratory-based studies during the development of new commercially relevant toxins such as ARP150v02.

## Results

### Characteristics of a modified Vip3C protein used in these studies

**Figure 1:**
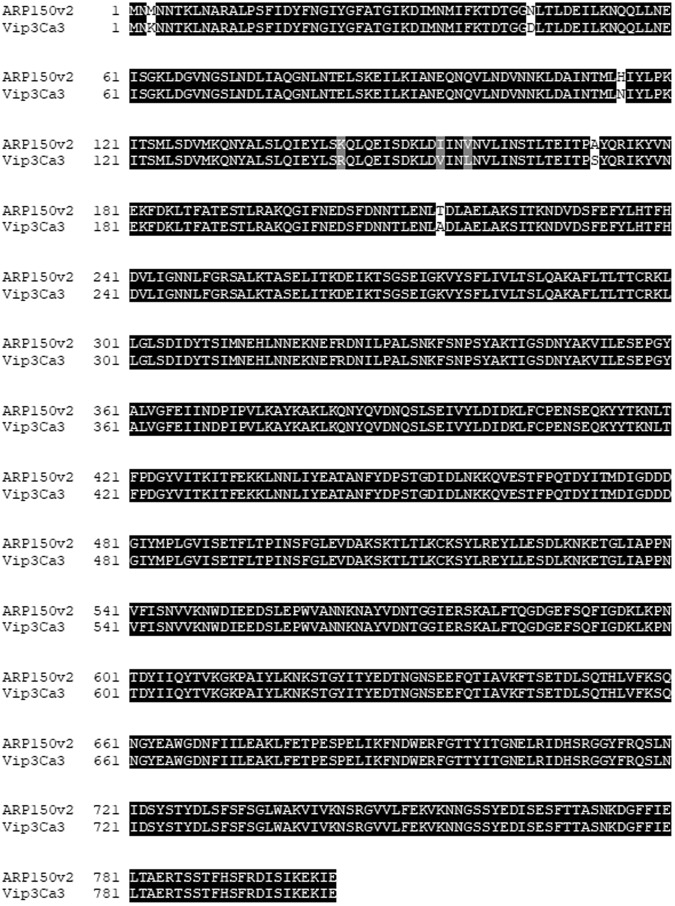
ARP150v02 sequence alignment with Vip3Ca3. White indicates non-conservative differences, and grey indicates conservative differences.

The amino acid sequence of ARP150v02 differs from Vip3Ca3 at 8 locations near the N-terminus (Fig. 1). The protein was recombinantly expressed and purified from an *E*. *coli* vector with an N- terminal 6X His tag, and was tested in bioassays to assess its efficacy and spectrum of activity against agronomically important lepidopteran pests. ARP150v02 showed activity against a number of pests, causing mortality and or stunting in *Spodoptera exigua*, *S*. *frugiperda*, *Ostrinia nubilalis*, and *Agrotis ipsilon* (Table 1). Separate bioassays were used to determine the LC_50_ of ARP150v02 against two insect species. Against *S*. *frugiperda* it showed an LC_50_ of 450 ng/cm^2^. However, this protein showed low activity against *H*. *armigera*, since it was not even able to cause growth inhibition at 6000 ng/cm^2^ when tested at Universitat de València (Table 2). Only when the protein was tested at a higher dose against *H*. *armigera* at CSIRO was activity seen. To test whether ARP150v02 might overcome resistance to Vip3A in insects, the protein was tested against a colony of *H*. *armigera* that is resistant to Cry2Ab and Vip3Aa, called Ha-DRES, and against a colony susceptible to those toxins, called Ha-Gr. ARP150v02 killed approximately two-thirds of the susceptible insects at a dose of 10000 ng/cm^2^ and had an LC50 of 3560 ng/cm^2^, but had little to no effect on the resistant insects at 10000 ng/cm^2^ (Table 3), showing that the protein cannot be used to overcome resistance of *H*. *armigera* to Vip3Aa.

**Table 1:** Spectrum of activity of ARP150v02 showing semiquantitative activity against several insect species.

	0.01 µg/m		40 µg/ml	
	Mortality	Stunting		Stunting
*Spodoptera exigua*	0%	−	75%	++++
*Spodoptera frugiperda*	0%	++	50%	++++
*Ostrinia nubilalis*	0%	+	0%	+++
*Agrotis ipsilon*	0%	−	0%	+++

For stunting, a value of (−) indicates no stunting and a value of (++++) indicates the larvae remained the size of neonates.

**Table 2:** Quantitative assay of ARP150v02 insecticidal activity against *S*. *frugiperda* and *H*. *armigera*.

	LC_50_ (ng/cm^2^)	FL_95_ (ng/cm^2^)	slope ± SE
*S*. *frugiperda*	450	300–670	1.2 ± 0.1
*H*. *armigera*	>6000^a^	—	—

^a^ No mortality or growth inhibition was observed at this concentration.

**Table 3:** ARP150v02 insecticidal activity against *H*. *armigera* strains, susceptible (Ha-Gr) and resistant to both Cry2Ab and Vip3Aa proteins (Ha-DRES).

	LC_50_ (ng/cm^2^)	FL_95_ (ng/cm^2^)	slope ± SE
Ha- Gr	3560	2500–5420	1.20 ± 0.09
Ha-DRES	>10000^*^	—	—

^*^Little to no mortality was observed at this concentration.

### Binding of ^125^I-labeled ARP150v02 to BBMV

**Figure 2:**
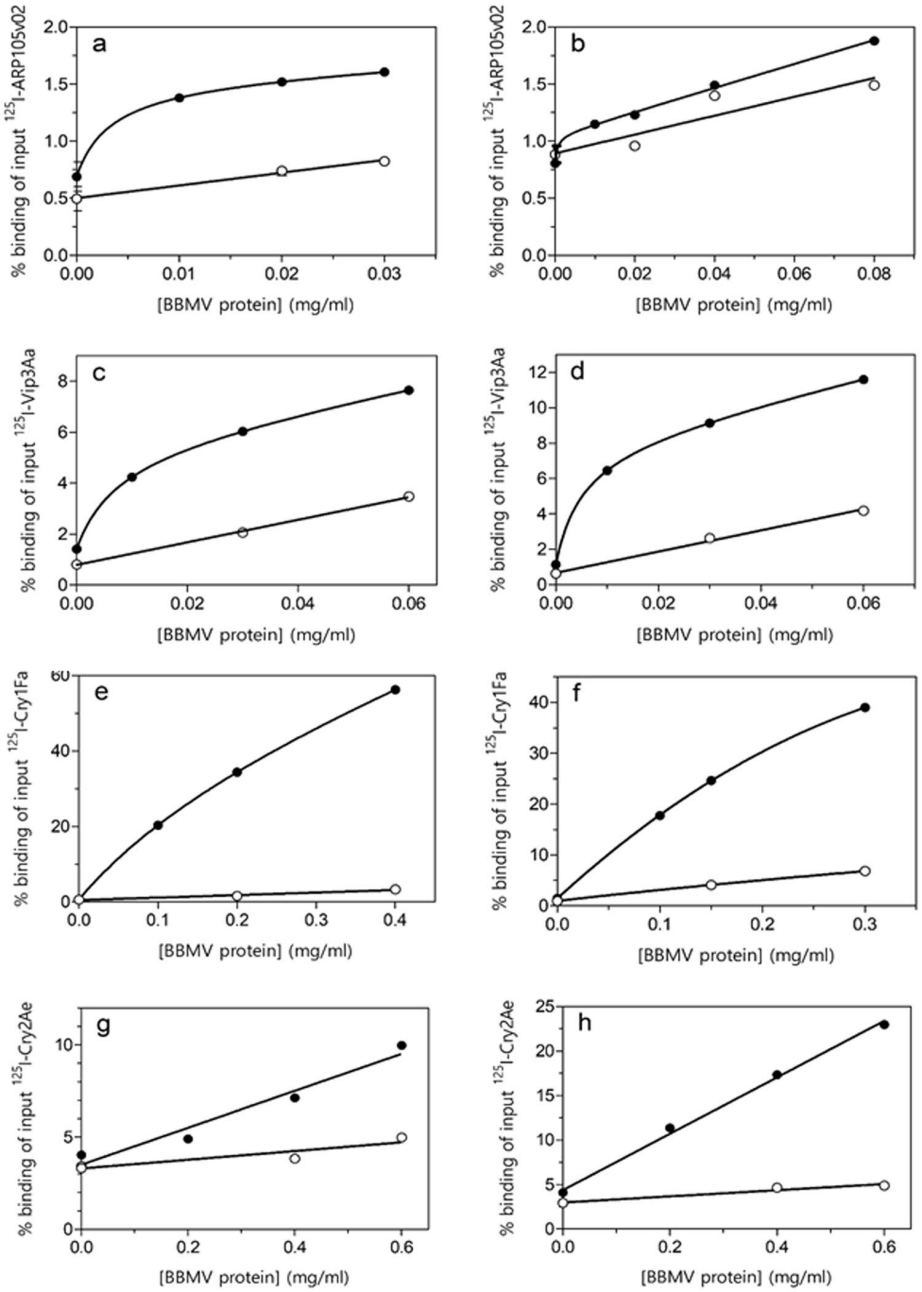
Binding assays at increasing concentrations of BBMV proteins. (**a**,**c**,**e**,**g**) show results for *S*. *frugiperda* BBMV. (**b**,**d**,**f**,**h**) show results for *H*. *armigera* BBMV. (•) total binding, (○) nonspecific binding.

**Figure 3:**
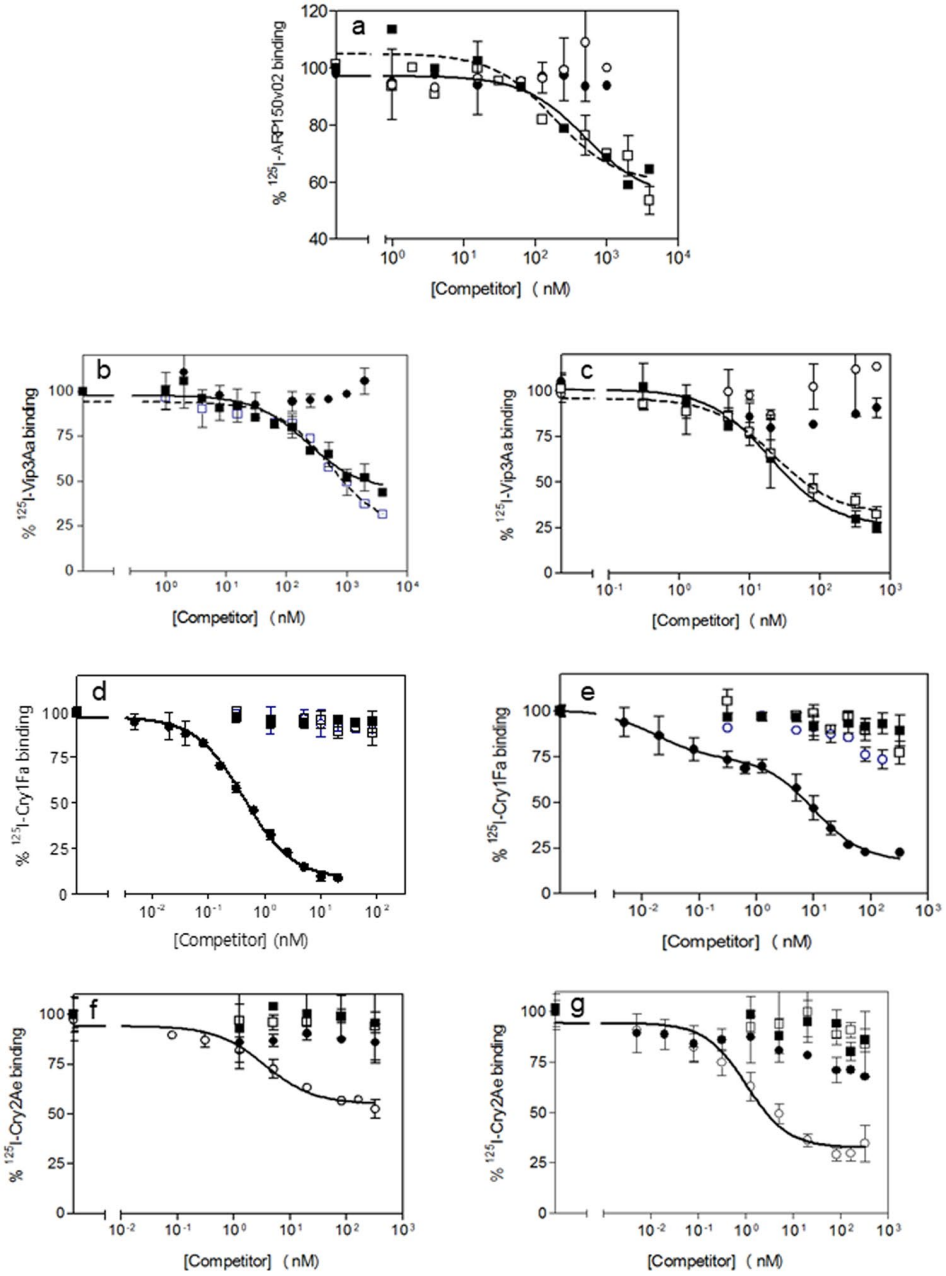
Competition binding assays with BBMV at increasing concentrations of unlabeled competitors. (**a**,**b**,**d**,**f**) show results for *S*. *frugiperda* BBMV. (**c**,**e**,**g**) show results for *H*. *armigera* BBMV. (•) Cry1Fa, (○) Cry2Ae, (■) Vip3Aa, (□) ARP150v02. Each data point represents the mean of at least two replicates and the error bars represent the standard error.

Total binding and nonspecific binding of ARP150v02 to BBMV from *S*. *frugiperda* and *H*. *armigera* is shown in Fig. 2a,b. The total binding was very low (around 1.5% at 0.03 mg/ml) with around 50% nonspecific binding in the case of *S*. *frugiperda* and almost 80% in the case of *H*. *armigera*. Since the binding was low and since the protein shows low toxicity to *H*. *armigera*, no further work was done with BBMV from this species. The increase of specific binding reached the asymptotic point at a very low concentration of *S*. *frugiperda* BBMV, for which 0.02 mg/ml BBMV were used in the competition assays (less than that made the pellet in the binding reaction too loose so it was easily lost in the washings). Heterologous competition binding assays (competition of a labeled protein by a different unlabeled protein) showed that Vip3Aa, but not Cry1Fa or Cry2Ae, competed for ARP150v02 binding sites (Fig. 3a). The dissociation constant (*K*_*d*_) and concentration of binding sites (*R*_*t*_) were estimated from the homologous competition assay (competition of a labeled protein by the same unlabeled protein) (Fig. 3a, solid line). The homologous competition curve fit a one-site model and the estimated *K*_*d*_ value indicated that the binding was of low affinity (Table 4).

**Table 4:** Binding parameters, *K*_*d*_ (dissociation constant) (nM) and *R*_*t*_ (concentration of binding sites) (pmol/mg), for the Vip3 and Cry proteins estimated from the respective homologous competition assays with BBMV from *S*. *frugiperda* and *H*. *armigera*.

Protein	Mean ± SE					
	*S*. *frugiperda*		*H*. *armigera*^a^			
	*K*_*d*_	*R*_*t*_	*K*_*d1*_	*R*_*t1*_	*K*_*d2*_	*R*_*t2*_
ARP150v02	103 ± 55	48 ± 39	—	—	—	—
Vip3Aa	17 ± 5	50 ± 11	54 ± 10	138 ± 23	—	—
Cry1Fa	0.30 ± 0.02	1.30 ± 0.05	0.045 ± 0.003	0.023 ± 0.014	6.0 ± 0.3	14.1 ± 0.2
Cry2Ae	3.4 ± 0.6	0.26 ± 0.04	1.19 ± 0.03	0.40 ± 0.03	—	—

^a^Two binding sites were obtained for Cry1Fa in this insect species.

### Binding of ^125^I-labeled Vip3Aa to BBMV

Figure 2c,d show the binding of Vip3Aa to BBMV from the two insect species. The total binding was low (5–8% at 0.02 mg/ml of BBMV) with a substantial contribution of nonspecific binding (around 50%). As with labeled ARP150v02, the increase of specific binding reached the asymptotic point at a very low concentration of BBMV in both cases. As a consequence, the competition assays were performed with 0.02 mg/ml. Competition assays showed that ARP150v02, but not Cry1Fa (Cry2Ae was not tested), competed for Vip3Aa binding sites (Fig. 3b,c). The homologous competition curves fit a one-site model and their analysis indicated that the binding was of relatively high affinity in both insect species (Table 4).

### Binding of ^125^I-labeled Cry1Fa to BBMV

Total and nonspecific binding of labeled Cry1Fa to BBMV from *S*. *frugiperda* and *H*. *armigera* is shown in Fig. 2e,f. Approximately 40% of the labeled protein bound to BBMV (at 0.3 mg/ml of BBMV), with very low nonspecific binding contribution. A concentration of 0.1 mg/ml of BBMV was chosen to carry out the competition assays. The statistical analysis of the homologous competition curves indicated that with *S*. *frugiperda* BBMV the curve fit a one-binding site model, whereas with *H*. *armigera* BBMV the curve fit a two-binding site model (Fig. 3d,e). Interestingly, the lower affinity binding site in *H*. *armigera* was not detected when using preparations of BBMV that had been kept at −80 °C for more than two months (data not shown). In both insect species, the *K*_*d*_ values indicate that binding of Cry1Fa is of high affinity (Table 4).

Heterologous competition binding assays showed that neither ARP150v02, Vip3Aa or Cry2Ae competed with Cry1Fa for binding to the BBMV from both insect species (Fig. 3d,e), indicating that the Cry1Fa binding site (or sites, if there was more than one site with similar *K*_*d*_ values) is not shared with the other three proteins.

### Binding of ^125^I-labeled Cry2Ae to BBMV

Total and nonspecific binding of labeled Cry2Ae to BBMV from the two insect species is shown in Fig. 2g,h. The total binding with *S*. *frugiperda* BBMV was relatively low (10% at 0.6 mg/ml of BBMV) and more than 50% was due to nonspecific binding (Fig. 2g). However, with *H*. *armigera* BBMV almost 25% total binding was obtained (at 0.6 mg/ml of BBMV) with a very low contribution of nonspecific binding (Fig. 2h). Competition binding assays were carried out with 0.4 mg/ml of BBMV. The results showed that neither ARP150v02, Vip3Aa, or Cry1Fa competed for Cry2Ae binding sites (Fig. 3f,g). The homologous competition curves fit a one-site model and their analysis indicated that the binding was of high affinity in both insect species (Table 4).

### Field Activity of ARP150v02

**Figure 4:**
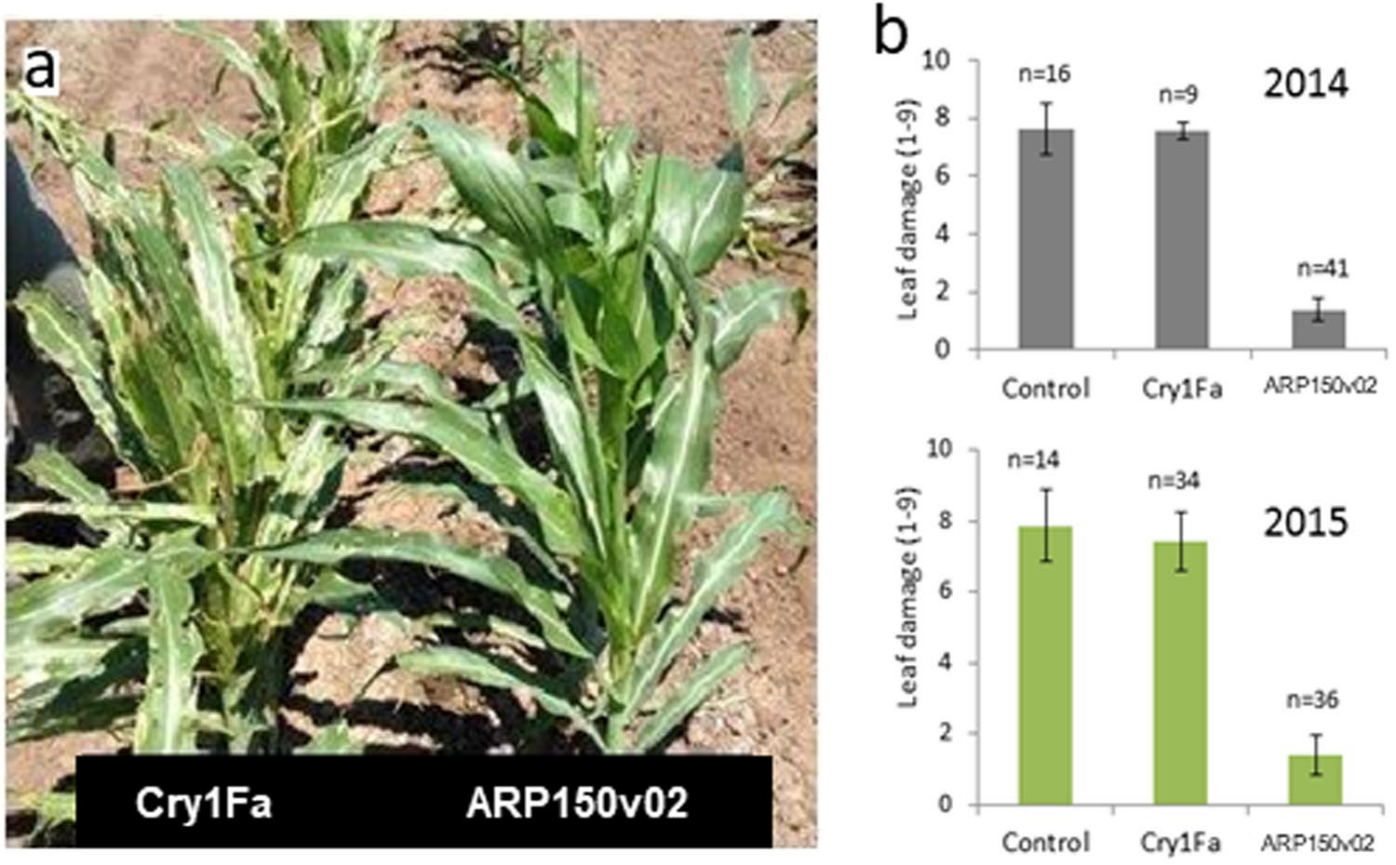
Field performance of corn plants expressing ARP150v02. (**a**) Transgenic corn plants expressing ARP150v02 (right) compared to transgenic plants expressing Cry1Fa (left). (**b**) Average leaf damage scores from field-tested transgenic corn plants, on a scale from 1 to 9, where 1 means no visual damage and 9 means the leaves are almost totally destroyed. The number of transgenic events tested is indicated. Control plants were nontransgenic.

Field trials were conducted in 2014 and 2015 in a region where a natural infestation of Cry1Fa-resistant *S*. *frugiperda* is known to persist^21^ to judge the commercial potential of ARP150v02. To confirm the presence of the Cry1Fa-resistant population, transgenic maize plants expressing Cry1Fa were planted alongside plants expressing ARP150v02 protein. Significant leaf damage was observed in Cry1Fa-expressing plants as well as in negative control plants (not shown in photo), but plants expressing ARP150v02 protein were protected (Fig. 4a). Leaf damage was measured on a 1–9 scale, with 1 representing no visible damage or only pinhole sized lesions, and 9 representing nearly complete destruction of whorl and furl leaves. In both 2014 and 2015, *S*. *frugiperda* pressure was high as indicated by the high leaf damage scores of 7.6 and 7.8 respectively in the negative control plants (Fig. 4b). Maize plants expressing Cry1Fa also demonstrated high leaf damage scores of 7.5 and 7.4 (2014 and 2015 respectively) indicating the presence of the Cry1Fa-resistant insect population. However, maize plants expressing the modified Vip3C exhibited excellent control of the resistant *S*. *frugiperda* population as seen by the relatively low leaf score of 1.4 in both years (Fig. 4b).

## Discussion

Previous studies on Vip3Aa provided LC_50_ values of 620 ng/cm^2^ for *S*. *frugiperda*^22^ and 1660 ng/cm^2^ for *H*. *armigera*^23^, while Vip3Ca was much less toxic to both insect species: a concentration as high as 4000 ng/cm^2^ of Vip3Ca3 killed 27% of neonate *S*. *frugiperda* larvae and 65% of neonate *H*. *armigera* larvae after 10 days^24^. Despite the low toxicity reported for Vip3Ca3 against *S*. *frugiperda*, in the current study ARP150v02, which differs from Vip3Ca3 in eight amino acid positions near the N-terminus, showed substantial activity against this insect pest, with an LC_50_ value of 450 ng/cm^2^ (Table 2), not significantly different from the value obtained for Vip3Aa. Thus ARP150v02 can potentially be substituted for Vip3Aa for pest control in crops though, like Vip3C, it will not control *H*. *armigera* that have developed resistance to Vip3Aa. The amino acid differences between ARP150v02 and Vip3Ca3 led to a reduction in the already low activity that Vip3 proteins exhibit against *H*. *armigera*^3^, suggesting the possibility that modifications that increase effectiveness against some pests may lead to loss of activity against other insect species.

The fact that ARP150v02 does not kill Vip3Aa-resistant *H*. *armigera* suggested that it might share binding sites with Vip3Aa, but few competition binding experiments comparing Vip3A and Vip3C proteins have previously been done. This study represents the first time a member of the Vip3C protein family has been radiolabeled, improving the determination of quantitative binding parameters with insect BBMV so that competition between ARP150v02 and Vip3Aa could be directly assessed. The high *K*_*d*_ value for ARP150v02 with *S*. *frugiperda* BBMV (103 nM) indicates that binding of this protein is of low affinity. The *K*_*d*_ value for *H*. *armigera* BBMV could not be estimated due to the extremely low specific binding and high nonspecific binding obtained. Heterologous competition assays showed that ARP150v02 and Vip3Aa share binding sites in *S*. *frugiperda*, whereas the Cry proteins used in this study do not recognize the Vip3 binding sites, consistent with the results of the insect bioassays and field studies.

Vip3Aa binding to *S*. *frugiperda* BBMV can be considered to be of high affinity, though the *K*_*d*_ is significantly higher than that of Cry1Fa or Cry2Ae (Table 4). The *K*_*d*_ value obtained is essentially the same as the one reported in a previous study using a different source of protein (Vip3Aa16)^25^. For *H*. *armigera* this is the first time that a *K*_*d*_ value has been obtained for a Vip3 protein. The high *K*_*d*_ value (54 nM) indicates that binding of Vip3Aa to *H*. *armigera* BBMV is of low affinity. Although the relationship between binding affinity and insecticidal potency is not always direct^26^, in our case the lower affinity of Vip3Aa to *H*. *armigera*, as compared with *S*. *frugiperda*, is correlated with the lower susceptibility of *H*. *armigera* to this protein. The same is true for ARP150v02, with its affinity for BBMV from the two insect species correlating with its activity against those species. The results from the heterologous competition assays extend those previously obtained with biotin-labeled Vip3Aa and Vip3Af, in that Cry2Ab (with *Heliothis virescens* BBMV) and Cry1Fa (with *S*. *frugiperda* BBMV) do not compete for Vip3A binding sites^19,27^. In contrast, ARP150v02 does compete for Vip3Aa binding sites in *S*. *frugiperda* and *H*. *armigera* (Fig. 3a–c), a result that is consistent with one recently obtained for Vip3 proteins with *Mamestra brassicae*^20^.

The homologous competition experiments with Cry1Fa indicate that it binds with very high affinity to BBMV from both insect species (Table 4), in agreement with previous studies^28,29^. The lower *K*_*d*_ values for *S*. *frugiperda* obtained in the present study compared to earlier ones^17,28,29^, and the finding of two binding sites in *H*. *armigera*, may be due to the use of more highly purified protein and fresh BBMV. The results from the heterologous competition experiments are in agreement with previous studies that showed that Vip3A and Cry2A proteins do not bind to Cry1Fa binding sites in *S*. *frugiperda*^19,28^ and *Helicoverpa zea*^18^.

Cry2Ae also bound with high affinity to BBMV from the two species (Table 4). This is the first time that the *K*_*d*_ value of this protein to *S*. *frugiperda* BBMV has been reported. For *H*. *armigera*, the *K*_*d*_ value obtained (1.19 nM) is in good agreement with the ones previously reported for Cry2Ae and for Cry2Ab in *H*. *armigera* and other heliothine species (*K*_*d*_ values between 2.3 and 6.5 nM)^11,15,18^. The results from the heterologous competition experiments extend those obtained with another heliothine species, *H*. zea, in that the Cry2Ae binding site is not shared by Cry1Fa or Vip3Aa^18^.

**Figure 5:**
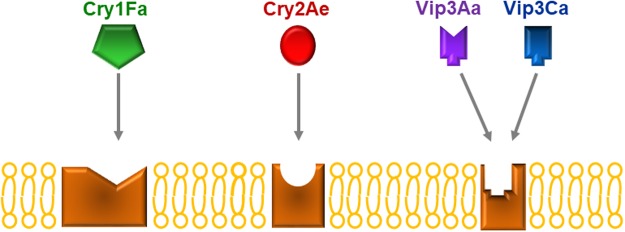
Binding site model for the Cry and Vip3 proteins in this study for *S*. *frugiperda* and *H*. *armigera*.

Taking all the heterologous competition results together we can propose a binding site model for the four *B*. *thuringiensis* proteins and the two insect species in this study (Fig. 5), in which Cry1Fa and Cry2Ae each bind to independent binding sites, not shared by the Vip3 proteins, and that Vip3Aa and ARP150v02 bind to a common binding site not shared by the Cry proteins. According to this model, the choice of one of these Vip3 proteins to be expressed in a Bt crop should be made on the basis of differences in their spectrum of activity, but not as a solution for resistance management.

The competition binding results fit together with the laboratory bioassays using insects resistant to both Cry2 and Vip3A. ARP150v02 was active against susceptible *H*. *armigera*, but was inactive against a colony that was resistant to Vip3A proteins. We did not determine if the cross-resistance was due to loss of binding, or due to a change in a subsequent step in the toxicity process. Binding experiments with BBMV isolated from resistant insects would be one way to address this question. In addition to being in agreement with the laboratory insect bioassays, the binding results are also in agreement with field trials using maize expressing Cry1Fa or ARP150v02. *Spodoptera frugiperda* that were resistant to Cry1F did extensive damage to plants expressing Cry1F, but did minimal damage to plants expressing ARP150v02. This is the first time a full set of experiments has been used to connect competition binding experiments, insect bioassays, and field trials in one study. This agreement between laboratory *in vitro* binding experiments, laboratory insect bioassays, and field trials confirms the utility of the laboratory experiments in predicting the performance of transgenic crops in the field, at least for the case of ARP150v02, a fact that could be useful in the future development of commercial insect control traits. Performing binding experiments is typically much quicker and easier than obtaining resistant insect colonies, so being able to use binding experiments to predict the resistance management potential of a trait could be of great value.

The differences in amino acid sequence between ARP150v02 and other Vip3Ca proteins are located near the N-terminus of the protein. These differences increased activity against *S*. *frugiperda* and decreased activity against *H*. *armigera*, showing that the N-terminal region of the protein plays a role in the spectrum of activity. However, ARP150v02 still competes with Vip3Aa for the same binding sites in *S*. *frugiperda* BBMV, indicating that the changes in activity are not due to changes in the site of action of the protein. The differences in activity may be due to other factors, such as the stability of the protein in the insect gut, binding affinity, or steps in the intoxication process that come after receptor binding.

## Materials and Methods

### Expression and purification of B. thuringiensis proteins

Cry2Ae (accession number AAQ52362), Vip3Aa1 (accession number AAC37036) and ARP150v02 were produced from *Escherichia coli* BL21 and Cry1Fa (accession number AAA22348) from *E*. *coli* WK6. *Escherichia coli* cells were grown in TB-medium for three to four hours at 37 °C with shaking until reaching an OD_600_ between 0.6 and 0.9. Then, the culture was made 1 mM IPTG and incubated for 18 h to induce the expression of the recombinant proteins. The culture medium was centrifuged at 9000 *g* for 30 min at 4 °C to proceed with the lysis.

**Figure 6:**
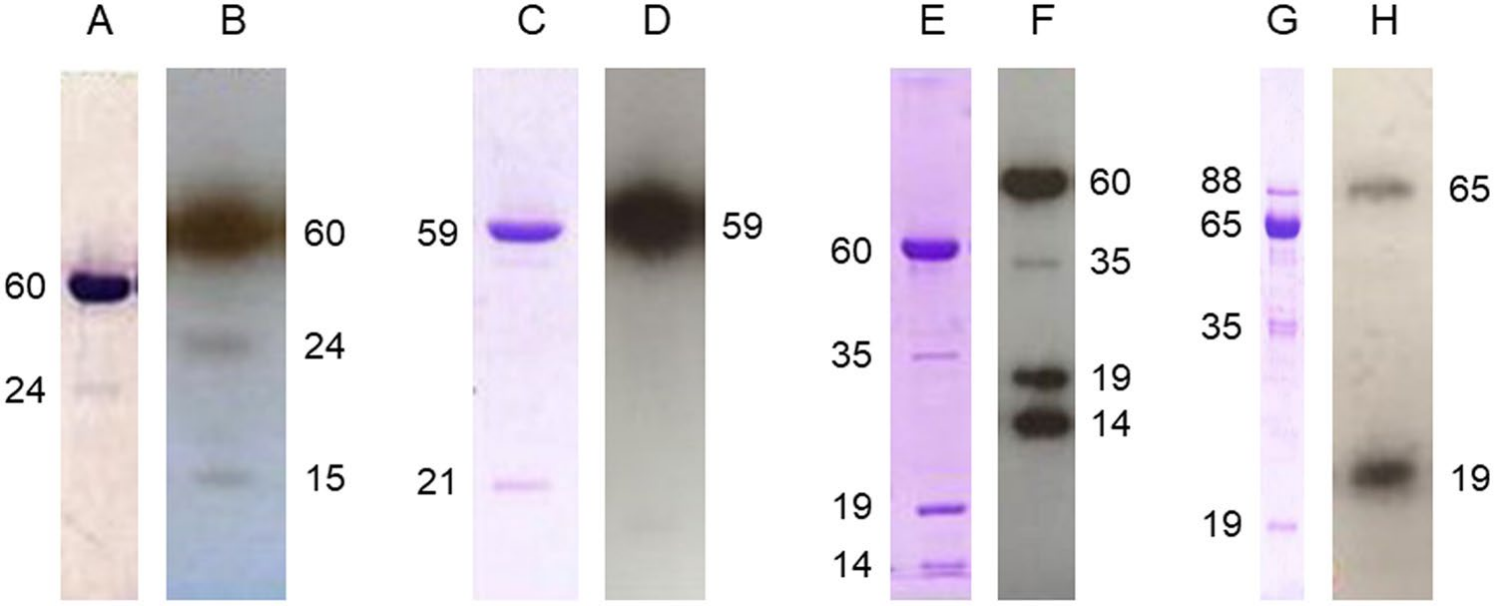
SDS-PAGE analysis of the purity of the protein preparations used for labeling as revealed by Coomassie blue (**A**,**C**,**E**,**G**) or autoradiography (**B**,**D**,**F**,**H**). (**A**,**B**) Cry1Fa; (**C**,**D**) Cry2Ae; (**E**,**F**) Vip3Aa; (**G**,**H**) ARP150v02. Numbers on the side refer to the approximate molecular weight of the main bands. The figure shows full length lanes of SDS-PAGE analyses performed separately.

For Cry1Fa purification, the pelleted cells were resuspended in lysis buffer (50 mM Tris-HCl, 5 mM EDTA, 100 mM NaCl, pH 8) with vigorous vortexing and then lysed by 2 min sonication (10 sec ON, 10 sec OFF). After centrifugation, the insoluble material (containing the Cry1Fa inclusion bodies) was washed three times with lysis buffer and then solubilized in carbonate buffer (50 mM sodium carbonate, 100 mM NaCl, 10 mM DTT, pH 10.5) for 2 h at 37 °C. The solubilized protein was activated with 10% trypsin for 1 h at 37 °C and then dialyzed at 4 °C against 20 mM Tris/HCl (pH 8.6). The activated Cry1Fa was further purified on a HiTrap Q HP column (5 ml bed volume) equilibrated in the same dialysis buffer, using an Äkta explorer 100 system (GE Healthcare, UK). Cry1Fa was eluted with a linear gradient of 1 M NaCl (0–80% in 100 ml). The fractions containing Cry1F were pooled and DTT was added at a final concentration of 10 mM. After 5 min incubation at room temperature, the mixture was loaded onto a gel filtration column (Superdex 200 10/300 GL, GE Healthcare, UK) pre-equilibrated with 20 mM Tris, 150 mM NaCl, 10 mM DTT, pH 8.6. The most concentrated peak fractions containing Cry1F (Fig. 6) were pooled and the DTT was eliminated by overnight dialysis (with one buffer change) at 4 °C against 20 mM Tris, 150 mM NaCl, pH 8.6.

For Cry2Ae (which carried a his-MBP tag at the N-terminus), the pelleted cells were resuspended in lysis buffer (50 mM sodium carbonate, 200 mM NaCl, 1 mM DTT, pH 8.9) and 800 µg of lysozyme and 0.2 mg of DNase was added per gram of pellet and incubated at 37 °C. Lysis was completed by 2 min sonication (10 sec on, 10 sec off). The supernatant (after centrifugation) was loaded onto a 5 ml MBP affinity column (MBPTrap HP from GE Healthcare Life Sciences) pre-equilibrated in lysis buffer and then eluted in the same buffer with 10 mM maltose. Fractions containing Cry2Ae were pooled and activated with 4% trypsin overnight at 4 °C without agitation (this step released the his-MBP tag). The fractions containing Cry2Ae were pooled and DTT was added at a final concentration of 10 mM. After 5 min incubation at room temperature, the mixture was loaded onto a gel filtration column (Superdex 200 10/300 GL, GE Healthcare, UK) equilibrated in 50 mM carbonate buffer, 50 mM NaCl, 10 mM DTT, pH 8.9. The most concentrated peak fractions containing Cry2Ae were pooled and reloaded onto a HiTrap Q HP column equilibrated in 50 mM carbonate buffer, 50 mM NaCl, pH 8.9, in an Äkta Explorer 100 chromatography system. The activated Cry2Ae was recovered in the flow through (free from the his-MBP tag), concentrated using Centricon centrifugal filters (Millipore) and dialyzed overnight against 20 mM Tris pH 8.6, 150 mM NaCl, to eliminate the remaining DTT. The purified Cry2Ae protein was flash frozen and stored at −20 °C until used for radiolabeling (Fig. 6). The Cry2Ae protein used as unlabeled competitor in the binding assays was prepared as indicated before, except for the DTT treatment and the gel filtration chromatography step.

For Vip3Aa, the pelleted cells were resuspended in lysis buffer (20 mM phosphate buffer, 200 mM NaCl, pH 7.4) and lysed by 2 min sonication (10 sec on, 10 sec off). After centrifugation at 27000 *g* for 30 min and supernatant filtration through 0.20 µm filters, the soluble Vip3Aa protein in the cell lysate was purified by isoelectric point precipitation (Ipp). The pH 6.0 was empirically selected as the best pH for the Ipp because it yielded the purest Vip3Aa protein from *E*. *coli* BL21 lysate. The pH of the Vip3Aa-containing cell lysate was lowered by the dropwise addition of acetic acid until the pH was 6.0. After centrifugation, the precipitated protein was resuspended in 20 mM Tris, pH 9, and dialyzed against the same buffer. The dialyzed protein was divided into two aliquots. The aliquot which was meant to be used for labeling was further purified on a HiTrap Q HP column as described above. The fractions containing the purified protein were pooled and subjected to trypsin treatment with 1% trypsin (w/w) for 2 h at 37 °C (Fig. 6). The aliquot to be used as competitor in the binding assays was activated by trypsin, as above, without any further purification. Both protein samples were stored at −20 °C until used.

For ARP150v02 (which carried a his-tag at the N-terminus), the pelleted cells were resuspended in lysis buffer (PBS, 300 mM NaCl, pH 7.4) and lysed by 2 min sonication (10 sec on, 10 sec off). After centrifugation at 27000 *g* for 30 min and supernatant filtration through 0.20 µm filters, the soluble ARP150v02 protein was purified by means of a Hi-Trap chelating HP column (GE Healthcare) charged with Ni^2+^. The ARP150v02 sample was loaded onto a column equilibrated with 50 mM phosphate buffer, pH 8.0, containing 10 mM imidazole. After washing with 50 mM phosphate buffer, pH 8.0, with 40 mM imidazole, the bound proteins were eluted by the same buffer containing 200 mM imidazole. Fractions (1 ml) were collected in tubes containing EDTA to give a final concentration of 5 mM after collecting the eluate. Fractions containing the ARP150v02 protein were pooled, diluted to 1 mg/ml and dialyzed against 20 mM Tris, 300 mM NaCl, pH 9. The purified ARP150v02 protein was kept at −20 °C and used for bioassays at the University of València. For binding experiments, ARP150v02 was further activated with 24% of trypsin (w/w) and let stand for 3 days at 30 °C. Finally, the activated protein, without any further purification (Fig. 6), was kept at −20 °C until use.

For bioassays carried out at CSIRO with *H*. *armigera* susceptible or resistant to Cry2Ab and Vip3A, the Cry1Ac protein was produced from Bt strain HD73 as described in Akhurst *et al*.^30^. Cry2Ab toxin was produced from a clone of the *cry2Ab* gene of *B*. *thuringiensis* variety kurstaki HD-1 in *B*. *thuringiensis*. The original clone was provided by L. Masson (National Research Council, Montreal, QC, Canada). The concentration of toxin was estimated by scanning the gel and analyzing the density of the toxin band relative to a bovine serum albumin (BSA) standard using Scion Image 1.62 software (Scion Corporation, Frederick, MD). A Vip3Aa clone in *E*. *coli* was used as a source of toxin. Production and calibration of the Vip3Aa toxin was described in Mahon *et al*.^31^.

### Insect strains and bioassays

Laboratory colonies of *S*. *frugiperda* and *H*. *armigera* were reared on artificial diet and maintained at 25 ± 3 °C with 70 ± 5% RH and at 16/8 h (light/dark) photoperiod. The insecticidal activity of ARP150v02 was measured by a surface contamination assay using seven different concentrations of the protein (protoxin form). For each concentration and control, 16 neonate larvae of *S*. *frugiperda* and *H*. *armigera* were used. Serial dilutions of ARP150v02 protein were prepared either in PBS pH 7.4 (three replicates) or in 50 mM sodium carbonate, pH 10.5 (three replicates). Mortality was scored after 7 days. Since the mortality values were not significantly different in either buffer, the six replicates were combined and analyzed by probit analysis (POLO-PC, LeOra Software, 1987) to obtain the LC_50_ value (concentration that kills 50% of the insects).

In addition to the bioassays carried out with a *H*. *armigera* laboratory strain of insects originally collected from the field in Spain, the susceptibility of a Vip3Aa-susceptible laboratory strain and a double resistant strain of *H*. *armigera* from Australia was compared. The general laboratory strain designated GR is susceptible to Cry and Vip3A toxins. It was created in 2010 by bulk mating 5-6 individuals from 40 iso-female families that scored negative for Cry1Ac, Cry2Ab and Vip3A resistance in F_2_ screens. Details of the susceptible colony are provided in Mahon *et al*.^32^. The double resistant *H*. *armigera* colony (resistant to Cry2Ab and Vip3Aa) is designated DRES. It was created from individual resistance alleles isolated from the field as described in Walsh *et al*.^33^. The rearing methods were modified from those of Teakle and Jensen^34^, as described in Mahon *et al*.^35^ and Mahon *et al*.^36^. Bioassays with toxins were conducted using the surface contamination method as described for Cry2Ab assays^35^. Briefly, approximately 300 µl of standard diet was added diet to each well of plastic trays shaped with 96 wells sized so that the when containing diet, the upper surface accessible by larvae was approximately 0.57 cm^2^ in area. Once the diet had cooled, 0.2 ml of a solution containing an appropriate concentration of toxin was added and allowed to air dry. One neonate was placed in each well before it was sealed with a perforated heat-sensitive lid. Trays were incubated at 25 °C for 7 days by which time control larvae normally reach 3rd or 4th instar. Dead and alive larvae (capable of coordinated movement when prodded) were counted and the larval instars of surviving larvae were scored.

### Radiolabeling of Cry and Vip3 proteins

Iodination was performed using trypsin-activated proteins (25 μg) by the chloramine-T method^15,37^. Cry1Fa, Cry2Ae, and Vip3Aa were mixed with 0.5 mCi of ^125^I and 1/3 (vol/vol) of 18 mM chloramine-T. For ARP150v02, the amount of ^125^I was reduced to 0.125 mCi to prevent inactivation of the protein. The excess of free ^125^I was separated from the labeled protein using a Bio-Gel P-30 desalting column (165 × 1.7 mm inner diameter) (Bio-Rad) equilibrated in 20 mM Tris pH 8.6, 150 mM NaCl, 0.1% BSA. The purity of the ^125^I-labeled proteins was checked by analyzing the elution fractions by SDS-PAGE with further exposure of the dry gel to an X-ray film (Fig. 6). The specific activity of the labeled proteins was calculated based on the input toxin, the radioactivity eluting in the protein peak, and the percentage of radioactivity in the toxin band vs. that in minor bands as reveled by the autoradiography. The estimated specific activity of the labeled proteins was 31 mCi/mg for Cry1Fa, 38 mCi/mg for Cry2Ae, 2 mCi/mg for Vip3Aa, and 0.54 mCi/mg for ARP150v02.

### BBMV preparation

Last-instar larvae of *S*. *frugiperda* and *H*. *armigera* were dissected on ice-cold MET buffer (0.3 M mannitol, 5 mM EGTA, 17 mM Tris–HCl, pH 7.5). The midguts were carefully pulled out of the body carcass, stored in ice-cold MET buffer for no longer than 15 min, blotted on filter paper and then flash frozen in liquid nitrogen and stored at −80 °C. BBMV were prepared by the differential magnesium precipitation method^38^ and stored at −80 °C. The protein concentration in the BBMV preparations was determined by the Bradford method (1976) using BSA as standard.

### Binding assays

Binding assays were performed by incubating BBMV with radiolabeled protein (0.045 nM for Cry proteins and 1.2 nM for Vip3 proteins) at room temperature in a 0.1 ml final volume for different times and buffers depending on whether the labeled protein was Cry or Vip3. For labeled Cry proteins the incubation was carried out for 60 min in binding buffer A (8 mM Na_2_HPO_4_, 2 mM KH_2_PO_4_, 150 mM NaCl, pH 7.4, 0.1% BSA)^5,15^, whereas for labeled Vip3 proteins the conditions were 90 min and buffer B (20 mM Tris-HCl, 150 mM NaCl, 1 mM MnCl_2_, pH 7.4, 0.1% BSA)^25^. Prior to use, the buffer in which the BBMV were stored was exchanged to the appropriate binding buffer by centrifuging the BBMV sample for 10 min at 16000 *g* (in a refrigerated centrifuge) and resuspending the pellet in the binding buffer. The incubation was stopped by centrifuging the tubes at 16000 *g* for 10 min at 4 °C and the pellet was washed once with 500 µl of cold buffer. The radioactivity retained in the pellet was measured in a model 2480 WIZARD^2^ gamma counter. An excess of unlabeled protein (2000 ng) was used to estimate the non-specific binding. Specific binding was calculated by subtracting the non-specific binding from the total binding.

To determine the appropriate concentration of BBMV to use in competition assays, the labeled proteins were incubated with increasing concentrations of BBMV in the binding buffer. Competition assays consisted on the incubation of the labeled proteins with the appropriate concentration of BBMV (0.1 mg/ml for Cry1Fa, 0.4 mg/ml for Cry2Ae, 0.02 mg/ml for Vip3Aa and ARP150v02) (the same BBMV concentration was used for both insect species) at increasing concentrations of unlabeled competitor. Dissociation constant (*K*_*d*_) and concentration of binding sites (*R*_*t*_) were estimated using the LIGAND software^39^.

### Field trials

Field trials were conducted by DM Crop Research Group, Inc. at the Illinois Crop Improvement Farm in Juana Diaz, Puerto Rico. Each experimental unit consisted of a one-row plot of maize measuring approximately 12.5 feet. Plots were arranged using a randomized complete block design with four replications. Efficacy was determined by evaluating all positive plants in each plot. Traditional, non-GMO inbred B110 and a commercial hybrid were used as negative controls. A soil applied insecticide was applied at planting. In addition, foliar insecticides were applied up to the V4 leaf stage to control thrips and other early plant stage insect pests. Approximately twenty days after the last insecticide application, each plot was assigned a fall armyworm leaf damage rating (1–9; where 1 = no visual damage and 9 = leaves almost totally destroyed^40^. The damage was typical of *S*. *frugiperda*, with very large lesions. Egg masses and moths present at the time of leaf evaluation were all identified as *S*. *frugiperda*. No *Helicoverpa zea* or *H*. *armigera* larvae were observed feeding on leaves. A number of plants were dissected to verify that only *S*. *frugiperda* larvae were present.
